# Screening of Coronary Artery Origin by Echocardiography: Definition of Normal (and Abnormal) Take-Off by Standard Echocardiographic Views in a Healthy Pediatric Population

**DOI:** 10.3390/healthcare10101890

**Published:** 2022-09-28

**Authors:** Massimiliano Cantinotti, Pietro Marchese, Eliana Franchi, Alessandra Pizzuto, Giulia Corana, Cecilia Viacava, Benjamin T. Barnes, Shelby Kutty, Nadia Assanta, Colin J. McMahon, Martin Koestenberger, Raffaele Giordano

**Affiliations:** 1 Fondazione G. Monasterio CNR-Regione Toscana, Massa and Pisa, 54100 Massa, Italy; 2 Institute of Life Sciences, Scuola Superiore Sant’Anna, 56121 Pisa, Italy; 3 Blalock Taussig Thomas Heart Center, Johns Hopkins Hospital, Baltimore, MD 21205, USA; 4 Department of Paediatric Cardiology, Children’s Heart Centre, Children’s Health Ireland at Crumlin, 53354 Dublin, Ireland; 5 Division of Pediatric Cardiology, Department of Pediatrics, Medical University Graz, 8010 Graz, Austria; 6 Adult and Pediatric Cardiac Surgery, Department Advanced Biomedical Sciences, University of Naples “Federico II”, 80131 Naples, Italy

**Keywords:** echocardiography, coronary artery, children, athletes, screening

## Abstract

Background: Echocardiographic screening of anomalous coronary artery origin is of increasing interest for children participating in sport activities. However, criteria to define normal coronary artery origins in children are poorly defined. Thus, the aim of the present investigation is to define the normal origin and angle of emergence of coronary arteries by echocardiography in healthy children. Materials and methods: The distances of the left main and right coronary artery (LMCA, RCA) origins from the aortic annulus were measured in the parasternal long-axis view (LAX). The angle of coronary artery emergence was measured in the parasternal short-axis view (SAX). Results: A total of 700 healthy subjects (mean age: 9.53 ± 5.95 years; range: 1 day–17.98 years) were prospectively enrolled. The distance of the RCA and LMCA from the aortic annulus correlated with body surface area, and nomograms (Z-scores) were generated. The RCA origin was below the sinotubular junction (STJ) in 605 patients (86.43%), at the STJ in 66 patients (9.43%), and above the STJ in 29 patients (4.14%). The LMCA origin was below the STJ in 671 patients (95.86%), at the STJ in 12 patients (1.71%), and above the STJ in 17 patients (2.43%). With respect to the RCA, an emergence angle < 18.5° in the SAX predicted a high take-off. with a sensitivity of 98.3% and a specificity of 93.1% (AUC 0.998). With respect to the LMCA, an emergence angle > 119.5° in the SAX predicted a high take-off, with a sensitivity of 70.6% and a specificity of 82.4% (AUC 0.799). Conclusion: This study establishes nomograms for LMCA and RCA origin in standard echocardiographic projections in healthy children.

## 1. Background

There is an increasing demand for evaluation of coronary artery origin by echocardiography, especially among children and young adults undergoing preparticipation screening for sport activities. Anomalous aortic origin of the coronary artery (AAOCA) is a rare but potentially life-threatening condition, specifically for young athletes [1,2,3,4,5,6,7,8,9,10,11], representing the second most common cause of sudden cardiac death in young athletes [10,11,12]. Transthoracic echocardiography is the initial imaging modality for screening and diagnosis of AAOCA [1,2,3,4,5,6,7,8,9,10]; however, there is a lack of confidence in the feasibility and accuracy of echocardiography in detecting AAOCA and a lack of standardized protocols for the echocardiographic assessment of coronary arteries.

Protocols to be employed and essential findings to be searched for the screening of coronary arteries during echocardiography are limited, and the definitions and clinical significance of some defects (such as a high coronary artery take-off) remain controversial [10,11,12,13,14,15,16,17]. The definition of a normal coronary artery origin (i.e., the level at which a coronary artery should arise within the aortic sinuses) remains not well defined to date. Questions such as, “Where exactly should the normal coronary artery arise from the aorta, what is the range of normal and how to differentiate variations of normal from abnormal?” are subjects of constant debate.

The primary aim of this study is to define the normal origin of coronary arteries by standard echocardiographic views in a large population of healthy children. The secondary aim of the study is to determine the feasibility of imaging the coronary artery origins and the rate of AAOCA in a pediatric population.

## 2. Methods

Healthy Caucasian children evaluated in the outpatient department of the Pediatric Cardiology Department at Fondazione G. Monasterio CNR–Regione Toscana of Massa for screening of congenital heart disease and eligible for inclusion in the study were prospectively enrolled. The presence of innocent defects, such as a tiny patent ductus arteriosus with small or less left-to-right shunting observed in the first 3 days of life or a patent foramen ovale, were considered normal findings. Exclusion criteria included [18] evidence of congenital or acquired heart disease, known or suspected neuromuscular disease, genetic syndromes, chromosomal abnormalities, pulmonary hypertension, systemic hypertension, connective tissue disease, or family history of genetic cardiac disease. All patients underwent a complete two-dimensional examination, and images were digitally stored for subsequent offline analysis. Images were collected only in quiet and cooperative children. Infants were allowed to bottle feed and (for older children) to watch cartoons during the examinations. No child was sedated.

The study was approved by the local ethics committee, FTGM CE (approval code: Study “Bet” No. 390). Parents or legal guardians were informed and agreed to participate by providing written consent.

### 2.1. Echocardiographic Protocol

Images were obtained in the apical four-chamber view using an iE33 system (Philips Medical Systems, Bothell, WA, USA) with 8 and 5 MHz transducers and a Vivid E 95 system (GE Vingmed Ultrasound) with 6 and 5 MHz probes (GE Healthcare) and simultaneous electrocardiographic monitoring.

Coronary artery origins were evaluated in parasternal short-axis [10] and long-axis views [1,8,9]. The 5-chamber view was used to exclude retroaortic circumflex artery origin from the right coronary artery (RCA) and to assess left main coronary artery (LMCA) take-off in cases of unclear long-axis imaging. Parasagittal planes and short-axis views were used when there was suspicion of AAOCA [1,8,9]. Color Doppler with decreased color gain (e.g., 15–40 cm/s) was used to detect coronary flow in cases of suspected AAOCA [7,8].

**Figure 1:**
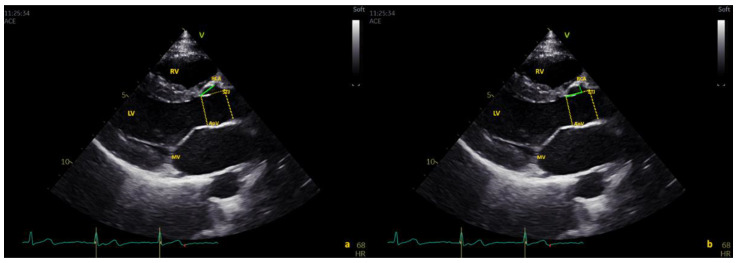
Measurement of right coronary artery (RCA) origin in long-axis view. (**a**) Coronary artery origin height measured using the oblique distance between the aortic annulus and proximal aspect of the coronary artery (**a**). The distance from the aortic annulus to the sinotubular junction (STJ) was measured, and the coronary artery height measurement was repeated on this horizontal line, tracing the perpendicular from the origin of the coronary ostium (**b**). LV = left ventricle, MV = mitral valve, RV = right ventricle, Aov =Aortic valve.

**Figure 2:**
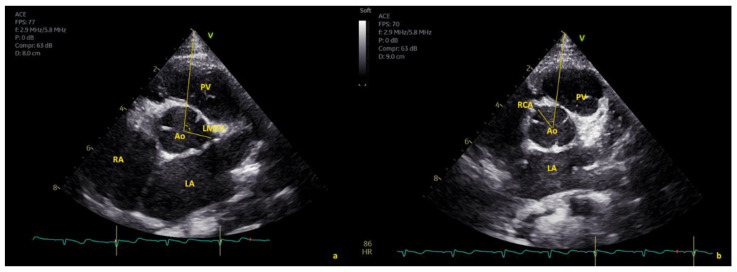
Angle measurement of LMCA (**a**) and RCA (**b**) origin in short-axis view. Ao = aorta, LA = left atrium, PV = pulmonary valve, RA = right atrium.

The distances of both the left and right coronary artery origins from the aortic annulus (AO) were measured in the parasternal long-axis view at end-systole. Coronary artery origin height was measured using the oblique distance between the aortic annulus and the proximal aspect of the coronary artery. The distance from the aortic annulus to the sinotubular junction (STJ) was measured from the aortic annulus to the STJ in end-systole. Coronary artery height measurement was then repeated on this horizontal line, tracing the perpendicular from the origin of the coronary ostium (Figure 1 and Figure 2). Dimensions of the aortic annulus, root, and sinotubular junction were also measured in end-systole from inner edge to inner edge, according to current pediatric guidelines [19]. A slit-like ostium was defined as ≥50% luminal narrowing of the ostium [11].

**Figure 3:**
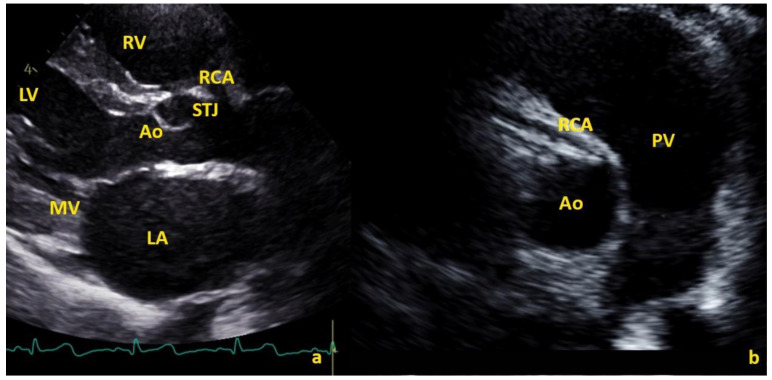
High RCA in long-axis view (**a**) and the acute angle and stilt-like ostium in short-axis view (**b**). Ao = aorta, LA = left atrium, LV = left ventricle, PV = pulmonary valve, RCA = right coronary artery, RV = right ventricle, STJ = sinotubular junction, MV = mitral valve.

The angle of origin of coronary arteries was measured in the parasternal short-axis (SAX) view (Figure 1, Figure 2 and Figure 3). The abscissa axis was delineated by taking the center of the aortic valve (i.e., the point where the three commissures join) as reference and tracing a line ending at the tip of the acoustic window (Figure 1, Figure 2 and Figure 3). Two experienced pediatric cardiologists (M.C. and E.F.) acquired the images and performed the measurements. Rates of intra- and interobserver variability were calculated from 20 randomly selected subjects.

Coronary artery height location was defined as the distance to the sinotubular junction (STJ) as follows:

- “Above the STJ” when the origin was >100% of the distance from the AO annulus to the STJ;
- “At the level of STJ” when the origin was within 5% of the distance from the AO annulus to the STJ; and
- “Below the STJ” when the value was <5% of the distance from the AO annulus to the STJ.

### 2.2. Statistical Methods

The sample size was calculated based on previous observations [18]. To examine the relationship of parameters of body surface area (BSA), heart rate (HR), and age with each of the echocardiographic variables, multiple models using linear, logarithmic, quadratic, cubic, power, exponential, and square root equations were tested [18]. Among the models that satisfied the assumption of homoscedasticity, the model with the highest R^2^ value was considered to provide the best fit. The presence or absence of heteroscedasticity, a statistical term used to describe the behavior of variance and normality of the residuals, was tested by the White test and the Breusch–Pagan test, as described previously [18]. To test the normality of residuals, Shapiro–Wilk and Lilliefors (Kolmogorov–Smirnov) tests were used [18]. Age, weight, height, HR, and BSA (by Haycock formula) [18] were used as independent variables in different regression analyses to predict the mean values of each echocardiographic measurement. Outliers to be excluded from analysis were identified visually and using the leverage values and the Studentized error residuals; observations were omitted in the final analysis if they significantly deviated from the models. The effect of sex was also evaluated as covariate in these models [18]. Intra- and interobserver variability of measurements were based on coefficient of variation (CV), and intraclass correlation coefficients (ICCs) in 20 subjects. The inter- and intra-CV were calculated as an average value based on the individual CVs for all duplicates. Inter-CVs of less than 15% are generally acceptable, whereas intra-CVs should be less than 10%.

Continuous variables are presented as mean ± SD and were compared using the Student t test; non-normally distributed variables are presented as median (interquartile range, IQR) and were compared using the Mann–Whitney U test. Categorical variables are expressed as frequency (%) and were compared with the chi-squared test. A two-tailed *p*-value < 0.05 was considered statistically significant. The Statistical Package for Social Sciences (SPSS) Release 23.0 (Chicago, IL, USA) and Stata Version 13 for Windows (Stata Corp, 2001) were used for analyses.

## 3. Results

Among the 720 children initially enrolled, 8 neonates and 12 infants were excluded as a result of inadequate images due to lack of cooperation. The final healthy cohort comprised 700 prospectively recruited subjects (age 1 day–17.98 years; 312 female). The mean age of the study population was 9.53 ± 5.95 years (median age: 9.45 years; interquartile range (IQR): 60–13.18 years). Cohort characteristics are detailed in Table 1.

### 3.1. Feasibility

Feasibility for coronary artery origin visualization was 100% for the left main coronary artery (including evaluation of bifurcation) and 98.8% for the RCA. In only one obese subject, it was not possible to visualize the RCA due to poor acoustic windows.

### 3.2. Percentage of Major and Minor Anomalies

Minor anomalies were found in 11 cases (1.57%), including 10 (1.42%) with separate ostial origin of the left anterior descending artery and left circumflex arteries (CFx) from the left coronary sinus and 1 (0.14%) coronary artery fistula from the LMCA to the main pulmonary artery. Major AAOCAs were found in six cases (0.86%), including three (0.43%) with a CFx originating from the right coronary sinus (posterior loop) and three (0.43%) with a single ostium coronary artery arising from the right coronary sinus. 

### 3.3. Coronary Artery Origin: Distance from the Aortic Annulus

#### 3.3.1. Nomograms

**Figure 4:**
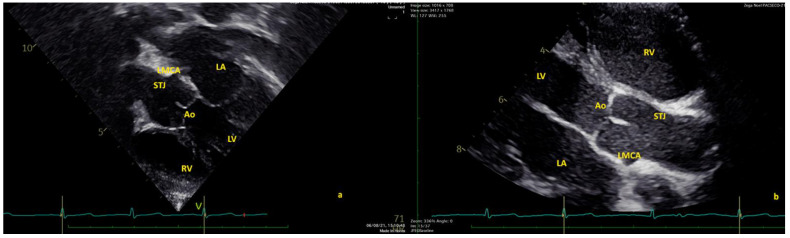
High left main coronary artery (LMCA) origin in modified four-chamber view (**a**) and in classical short-axis view; (**b**) angle of origin of the patient at 130° in short-axis view. Ao = aorta; LA = left atrium; LMCA = left main coronary artery; LV = left ventricle; STJ = sinotubular junction; RV = right ventricle.

The RCA and LMCA distances from the aortic valve showed a positive correlation with age, BSA, weight, and height (Supplemental Table S1). For all parameters, the association with BSA was stronger than with HR and age; thus, BSA was used for normalization of all parameters. The best-fit models (with the highest coefficient of determination, R^2^) for each measurement were exponential (ln[y] = a + b * ln[x]) (Supplemental Table S1). The predicted values and Z-score boundaries were derived using robust regression models, and cases with large absolute residuals were down-weighted (Supplemental Tables S2 and S3 and Figure 4). When the influence of sex was evaluated by multiple linear regression models incorporating sex as a covariate, along with BSA, no clinically significant effects were found (e.g., the maximum increase in R^2^ was 0.002, demonstrating that the additional contribution of sex in the models was marginal).

#### 3.3.2. Comparison of Measurement Methods

LMCA and RCA distances measured using the oblique distance between the aortic annulus and the proximal aspect of the coronary artery showed no significant differences compared to those obtained using a horizontal line tracing the perpendicular from the origin of the coronary ostium (*p* = 0.62 and *p* = 0.07, respectively). Thus, in the interest of simplicity, we decided to express and report only oblique measurements. In 50 subjects, the LMCA distance measurements were repeated in the parasternal long-axis and modified four-chamber views (when both were feasible). When compared, no significant difference emerged (*p* = 0.322). Again, in the interest of simplicity, we decided to express measurements interchangeably.

### 3.4. Coronary Arteries: Distance from STJ

#### 3.4.1. Right Coronary Artery (RCA)

Most of our healthy subjects (*n* = 605, 86.43%) had an RCA origin below the STJ, with a mean distance of 9.24 ± 3.59 mm/m^2^ from the aortic valve and of 4.56 ± 3.53 mm/m^2^ from the STJ in the long-axis view (LAX). In percentage terms, the RCA origin was located 60.45 ± 14.08% of the total distance from the annulus to the STJ within the aortic root. Subjects with an RCA origin below the STJ had a mean emergence angle of 35.73 ± 8.99° in the short-axis view (SAX).

Furthermore, 66 subjects (9.43%) had an RCA origin at the level of the STJ, with a mean distance of 11.34 ± 4.12 mm/m^2^ from the AoV and of 0.37 ± 0.25 mm/m^2^ from the STJ in the LAX; these subjects had a mean emergence angle of 30.97 ± 9.8° in the SAX.

**Figure 5:**
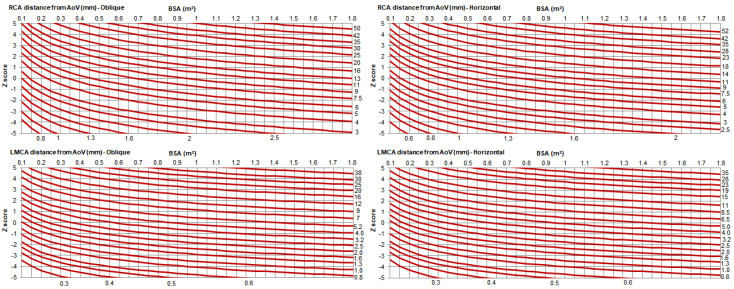
Predicted values and Z-score boundaries for RCA and LMCA. AoV = aortic valve, BSA = body surface area, LMCA = left main coronary artery, RCA = right coronary artery.

Finally, 29 (4.14%) subjects had an RCA origin above the STJ, with a mean distance of 1.32 ± 1.21 mm/m^2^. In these cases, the mean emergence angle in the SAX was 7.27 ± 14.06°. In eight of these cases, the RCA origin was >120% the distance from the Ao annulus to the STJ in the LAX. A slit-like origin was reported in five cases, with a significant luminal narrowing of the ostium of 52.5% to 57.65% (Figure 5). The remaining 24 cases exhibited mild luminal narrowing (mean: 23.76 ± 13.79 %; range: 3.33–47.18%).

#### 3.4.2. Left Main Coronary Artery (LMCA)

The origin of the LMCA was below the STJ in most subjects (*n* = 671, 95.86%), with a mean distance of 4.78 ± 2.38 mm/m^2^ from the aortic annulus and 8.68 ± 4.29 mm/m^2^ from the STJ in the LAX. In percentage terms, the LMCA origin was located 31.57 ± 12.73% of the total distance from the aortic annulus to the STJ in the LAX. Thus, the LMCA origin was most frequently proximally localized in the first third of the aortic root. Subjects with an LMCA origin below the STJ had a mean emergence angle of 111.97 ± 12.59° in the SAX.

In 12 (1.71%) healthy children, the LMCA origin was at the level of the STJ, with a mean distance of 10.03 ± 1.23 mm/m^2^ from the aortic annulus and 0.19 ± 0.14 mm/m^2^ from the STJ; these cases had a mean emergence angle of 131.15 ± 12.2° in the SAX.

In 17 (2.43%) healthy children, the LMCA origin was above the STJ, with a mean distance of 2.65 ± 4.3 mm/m^2^. In these cases, the mean emergence angle was 129.34 ± 18.45° in the SAX. In three of these cases, the LMCA origin was >120% of the distance from the Ao annulus to the STJ in the LAX. Among these 17 patients, none had a slit-like origin, whereas a minor luminal narrowing was present in all of these cases to varying degrees (mean: 22.14 ± 11.8%; range: 17.2–37.2%). Data are summarized in Table 2.

### 3.5. Correlation between Angle of Coronary Emergence in SAX and High Take-Off

Bivariate analysis revealed a positive correlation between RCA angle and RCA distance to the STJ expressed in absolute value and percentage (rho = 0.739 *p* < 0.001; rho = 0.752 *p* < 0.001, respectively). Analysis also revealed an inverse correlation between LMCA angle and LMCA distance to the STJ, either expressed in absolute value or percentage (rho = −0.411 *p* = 0.003; rho = −0.407 *p* = 0. 003, respectively).

#### 3.5.1. Right Coronary Artery (RCA): High Take-Off and Reduced Angle in SAX

Subjects with an RCA originating above the STJ demonstrated a significantly reduced angle of emergence compared with those originating below the STJ (mean 7.27 ± 14.06° vs. mean 35.46 ± 7.22° *p* < 0.001). An RCA emergence angle < 18.5° predicts an RCA with high take-off, with a sensitivity of 98.3% and a specificity of 93.1% (AUC 0.998).

Similarly, subjects with an RCA originating at the STJ demonstrated a significantly reduced angle of emergence in the SAX view compared with those originating below the STJ (mean 30.97 ± 9.80° vs. mean 36.31 ± 10.52°, *p* = 0.001). An RCA emergence angle < 27.85° predicts an RCA originating at the STJ, with a sensitivity of 86.8% and a specificity of 43.9% (AUC 0.646).

#### 3.5.2. Left Main Coronary Artery (LMCA): High Take-Off and Increased Angle in SAX

Subjects with a high take-off LMCA demonstrated a significantly increased angle of emergence in the SAX (*p* = 0.001) compared with those originating below the STJ (mean 129.34 ± 18.45° vs. mean 108.78 ± 20.37°, *p* = 0.001). An LMCA emergence angle > 119.5° in the SAX view predicts an LMCA originating above the STJ, with a sensitivity of 70.6% and a specificity of 82.4% (AUC 0.799).

Patients with an LMCA originating at the STJ demonstrated a significantly increased angle of emergence in the SAX compared with those originating below the STJ (mean 130.21 ± 11.30° vs. mean 113.84 ± 10.83°, Rho *p* < 0.001). An LMCA emergence angle > 118.45° predicts an LMCA originating at the STJ, with a sensitivity of 91.7% and a specificity of 75.00% (AUC 0.861) (Table 2).

### 3.6. Reproducibility

The inter- and intraobserver CV and ICC showed good reproducibility and an appropriate agreement among operators, as reported in Supplemental Table S4.

## 4. Discussion

The importance of evaluating coronary artery origin anomalies in competitive sport preparticipation screening is increasingly recognized, as AAOCA accounts for 15.7–19% of all causes of SCD in young athletes [10,11,12]. Many aspects of the evaluation of coronary artery origin by echocardiography remain unclear, including the definition of a normal origin by standard echocardiographic projections. We evaluated normal coronary artery origins by standard transthoracic echocardiography in a population of 700 prospectively enrolled healthy children. We also assessed the feasibility of imaging coronary artery origins and the detection rate of AAOCA using a multiprojection protocol. The major innovative aspect of the present investigation is the evaluation of the height of origin of coronary arteries within the aortic root measured in the parasternal long-axis view. The coronary artery angle of origin was systemically assessed in the short-axis view, and the relation between the angle of origin and height of origin was evaluated. Coronary artery origin visualization by echocardiography was previously found to be highly feasible in several studies [3,5,6,7,12,20]. We herein reported similar levels of feasibility for the RCA (98.8%) and the LMCA (100%).

We herein reported an incidence of major AAOCA of 0.86% (6 of 700 cases) and an incidence of minor anomalies of 1.57 % (11 of 700 cases). Our reported incidence of major AAOCA is similar to that published in autopsy series, i.e., 0.2–2.2% [12,13,14,15,16], and computed tomography (CT) series, i.e., 0.3–1.8% [15,16,17]. Interestingly, our reported incidence of major AAOCAs was higher than that previously reported in several echocardiographic studies. In these previous studies, the incidence of major AAOCAs considerably varied from 0.0% [5,12] to 0.09% [6], with the highest incidence of 0.76% [9]. It is possible that the lower incidence reported in these studies [5,6,12] was due to the use of imaging technologies less sensitive than that used in our study, especially with respect to the identification of small anatomical details, such as coronary arteries. Only one prospective study has been conducted to evaluate coronary artery origin, enrolling 1045 adolescents (12–15 years) [1]; however, to date, no large dataset is available in children younger than 12 years of age. In a study by Gerling et al. [1], the incidence of major AAOCA was reported to be 0.19%, whereas minor anomalies were identified in 1.5% of all cases. It is unclear whether the difference between our study results and those reported by Gerling et al. can be explained by the younger age group alone; interestingly, the incidence of minor anomalies was equivalent, at 1.5%.

Our study is the first echocardiographic approach to systematically evaluate the distance of a coronary artery from the aortic annulus and the sinotubular junction (STJ) in conventional parasternal long-axis view. As expected, the distance of origin from the aortic annulus was significantly related to age and body size. RCA origin was predominantly localized at 60.45 ± 14.08% of the distance from the aortic annulus to the STJ within the aortic root, whereas the LMCA was predominantly localized proximally in the first third of the aortic root. The site of origin of both the RCA and LMCA tended to migrate toward the STJ with somatic growth.

We report an incidence of RCA origin high take-off (above the STJ) in 29/700 (4.14%) of the healthy children investigated. The published incidence of high take-off in CT studies varies considerably among studies depending on the definition employed. When high take-off was defined by an origin >1 cm or >20% of the depth of the STJ, the incidence of RCA high take-off was 0.2%; conversely, when the cut-off was an origin above the STJ at any level, the prevalence of this defect increased to 0.36% [17]. Various echocardiographic studies [1,2,9] utilized the latter definition and reported an incidence of RCA origin high take-off ranging from 0.36% [9] to 1.14% [1]. There is some overlap between the incidence described in CT and echocardiography images. The outlier was that published by Gerling et al. [1], again in the largest prospective cohort, which is still less than that described herein. The difference, again, could be primarily due to our younger patient population.

Similarly, with respect to RCA, we herein report an incidence of high LMCA origin take-off of 17/700 (2.43%). LMCA origin high take-off is a rare defect with an estimated incidence ranging from 0.019% to 0.04% in retrospective cardiac catheterization studies [20,21,22,23,24,25]. Data sources for high LMCA take-off are practically absent for echocardiographic studies, making our current data of special interest and difficult to compare.

From a clinical point of view, the significance of a high origin or take-off of a coronary artery remains a controversial topic. There may be a risk of erroneously classifying benign variants as malignant anomalies potentially at-risk of sudden cardiac death (SCD) [17]. In the limited cases of reported SCD, this defect was associated with other coronary abnormalities, including hypoplastic coronary arteries, atherosclerotic disease, ostial stenosis, intramural tunneling, or a course between the aorta and pulmonary artery [23]. Studies have correlated higher coronary artery origin take-off with an increased risk of SCD [21,23]. Molossi et al. demonstrated [21] that three cases of high take-off (among 12,899 subjects, or 0.023%) described as the coronary artery originating >1 cm above the STJ were associated with an SCD. This use of adult criteria, i.e., 1 cm above the sinotubular junction, are unsuitable in children, as their aortic dimensions are smaller and constantly change with somatic growth. Thus, relative cut-off values (e.g., origin of 120% or more of the depth of the sinus of Valsalva or 20% or more the depth of the sinus above the STJ) were proposed for children [22]. In our series, only eight RCAs and three LMCAs had an origin >120% of the distance between the aortic annulus and the STJ. Whereas a mild luminal narrowing was noted in all high take-off group patients, only five RCA cases and no LMCA cases exhibited a real slit-like origin with a significant luminal narrowing of the ostium ≥ 50%. All 46 patients where incidentally discovered to have an origin of the RCA or LMCA at or above the STJ with a high take-off underwent additional investigations via Holter electrocardiogram and cycloergometer stress testing. All but one patient were found to be negative for exercise-induced myocardial ischemia. Ischemia was suspected in a single case upon stress test, which was confirmed by single-photon emission computed tomography (SPECT) with expression of an ischemic area in the perfusion territory of the RCA. Surprisingly, this case had an RCA origin that was not particularly high (0.89 mm/m^2^ or 1.8 mm above the STJ) nor an interarterial according to computed tomography (CT) scan, but a narrow angle of emergence of 8° was observed, with a slit-like orifice (narrowing of 57.6%), highlighting the importance of assessing the angle of emergence during routine echocardiographic screening.

An important finding of our prospective screening study is the relationship between the height of origin of coronary arteries in long-axis view and the angle of emergence evaluated in short-axis view. High RCA take-off was associated with a narrow angle of emergence. With respect to the RCA, an angle of emergence < 18.5° in short-axis view predicted a high take-off with very high sensitivity (98.3%) and specificity (93.1%) (AUC 0.998). In contrast, high LMCA take-off was associated with a wider angle of emergence. LMCA and an angle of emergence > 119.5° in short-axis view predicted a high origin, with a sensitivity of 70.6% and a specificity of 82.4% (AUC 0.799).

## 5. Limitations

Limited published data are available for comparison due to our young study population and the fact that the angle of emergence is not a well-studied measurement. High LMCA origin is often measured in a modified four-chamber view. However, we have proven, in a subset of children with a good acoustic window, that measurements of LMCA distance from the STJ obtained in long-axis and a modified four-chamber view did not show significant differences. Cases of high take-off were limited for the LMCA, which may limit the statistical power in this subset of subjects. As institutional practice, we perform SPECT or stress magnetic resonance imaging only in cases presenting with symptoms or signs leading to suspicion of ischemia upon cycloergometer stress testing [26]. The systematic use of these examinations, even in asymptomatic children with negative cycloergometer stress test, may be adopted to unmask silent ischemia in cases with very high take-off of coronary arteries. An ongoing clinical follow-up of cases with high RCA and LCA take-off will be helpful in the future to confirm the benign nature of these variants.

## 6. Conclusions

We herein report the height and angle of emergence of coronary artery origins evaluated by standard transthoracic echocardiography in a large population of healthy children. Angles of emergence in short-axis view and the height of origin from the annulus were strongly correlated. A cut-off value for an angle of emergence in short-axis view that could predict a high take-off of the coronary arteries was provided. These data may be helpful to ameliorate preparticipation screening for sport activities, the importance has been widely proven to prevent SCD in recent decades [27,28]. Systematic assessment of coronary artery origin anomalies by echocardiography currently not universally performed is a fast and easy method and should be included in preparticipation screening of in young athletes. Our data may be of use to echocardiographers in the rapid search for rare and subtle coronary anomalies, indicating potential risk of SCD [17], such as high take-off.

## Figures and Tables

**Table 1:** Study population.

*Variable*	*Male (n = 388)*	*Female (n = 312)*	*Total (n = 700)*
Demographic Data			
Age, years (range)	9.07 ± 4.97 (0–17.87)	8.21 ± 4.87 (0–17.98)	9.53 ± 5.95 (0–17.98)
Weight, kg (range)	36.59 ± 21.14 (2.57–110)	32.7 ± 19.03 (3–82)	36.62 ± 20.33 (2.57–110)
Height, cm (range)	132.33 ± 34.96 (11–196)	128.47 ± 33.51 (48–178)	130.7 ± 51.6 (46–196)
BSA Haycock, m^2^ (range)	1.14 ± 0.46 (0.18–2.43)	1.05 ± 0.45 (0.21–2)	1.1 ± 0.46 (0.18–2.43)
Anomalous aortic origin of coronary arteries			
LAD and CFx: separate ostia, nr	9	1	10
CFx from right coronary sinus, nr	3	0	3
Single ostium from the right coronary sinus, nr	2	1	3
Coronary artery fistula, nr	1	0	1
Coronary artery position within the aortic root			
RCA above the STJ, nr	15	14	29
RCA at the level of the STJ, nr	29	37	66
RCA below the STJ, nr	317	288	605
LMCA above the STJ, nr	8	9	17
LMCA at the level of the STJ, nr	6	6	12
LMCA below the STJ, nr	344	327	671

BSA = body surface area; CFx = left circumflex artery; LAD = left anterior descending artery; STJ = sinotubular junction; RCA = right coronary artery; LMCA = left main coronary artery.

**Table 2:** RCA position.

(A) RCA position within the aortic root in absolute value, percentage terms, and indexed by BSA.							
	**Mean**	**SD**	**Min**	**Max**	**Median**	**IQR 25th**	**IQR 75th**
RCA Below the STJ							
RCA distance from AoV (mm)—oblique	9.53	3.50	1.83	26.10	9.70	6.80	11.90
RCA distance from AoV (mm/m^2^)	9.24	3.59	2.27	29.53	8.73	6.92	10.78
RCA distance from AoV (%)	60.45	14.08	15.64	90.31	62.98	51.88	70.85
RCA distance from STJ (mm)	4.32	2.62	0.44	14.99	3.75	2.29	6.00
RCA distance from STJ (mm/m^2^)	4.56	3.53	0.56	27.72	3.70	2.07	5.85
RCA distance from STJ (%)	28.22	15.83	5.05	82.85	25.86	15.56	38.17
RCA diameter (mm)	2.46	0.82	0.60	7.50	2.34	1.90	2.97
RCA emergence degree (°)	35.73	8.99	9.7	116.6	34.75	30	40.78
RCA at the STJ							
RCA distance from AoV (mm)—oblique	12.21	2.78	5.47	18.02	12.40	10.90	13.70
RCA distance from AoV (mm/m^2^)	11.34	4.12	6.59	30.58	10.64	8.76	12.78
RCA distance from AoV (%)	79.66	5.50	68.18	98.47	80.09	76.67	83.01
RCA distance from STJ (mm)	0.40	0.25	0.00	0.94	0.40	0.20	0.60
RCA distance from STJ (mm/m^2^)	0.37	0.25	0.00	1.02	0.34	0.17	0.55
RCA distance from STJ (%)	2.61	1.48	0.00	4.82	2.74	1.48	3.92
RCA diameter (mm)	2.83	0.97	1.00	5.40	2.67	2.21	3.50
RCA emergence degree (°)	30.97	9.8	12.5	54.5	30.15	23.83	38.85
RCA above the STJ							
RCA distance from AoV (mm)—oblique	13.29	3.55	4.70	19.50	12.70	10.70	15.85
RCA distance from AoV (mm/m^2^)	12.28	3.42	6.63	20.63	11.40	9.85	13.27
RCA distance from AoV (%)	89.80	9.54	73.91	106.58	88.79	81.28	99.46
RCA distance from STJ (mm)	−1.40	1.36	−4.46	−0.01	−1.04	−2.29	−0.23
RCA distance from STJ (mm/m^2^)	−1.32	1.21	−4.27	−0.01	−0.90	−2.34	−0.17
RCA distance from STJ (%)	−10.02	9.20	−26.39	−0.07	−7.37	−19.88	−1.41
RCA diameter (mm)	3.01	0.95	1.36	4.90	3.00	2.43	3.79
RCA emergence degree (°)	7.27	14.06	−37.2	18	13.2	4.4	16.7
(B) LMCA position within the aortic root in absolute value, percentage terms, and indexed by BSA.							
LMCA below the STJ							
LMCA distance from AoV (mm)—oblique	4.86	2.35	1.00	16.80	4.42	3.24	5.70
LMCA distance from AoV (mm/m^2^)	4.78	2.38	1.08	16.55	4.14	3.18	5.64
LMCA distance from AoV (%)	31.57	12.73	8.04	93.71	28.50	23.26	36.34
LMCA distance from STJ (mm)	8.62	3.62	0.73	22.49	8.49	6.00	10.90
LMCA distance from STJ (mm/m^2^)	8.68	4.29	0.51	31.10	8.09	5.88	10.59
LMCA distance from STJ (%)	55.27	16.26	3.86	88.99	56.54	47.74	66.15
LMCA diameter (mm)	2.68	0.88	0.76	6.00	2.60	2.00	3.21
LMCA emergence degree (°)	111.97	12.59	11.60	163.00	113.00	105.00	120.00
LMCA at the STJ							
LMCA distance from AoV (mm)—oblique	14.65	2.22	11.60	17.90	14.45	12.80	16.58
LMCA distance from AoV (mm/m^2^)	10.03	1.23	8.48	12.33	9.85	8.99	10.83
LMCA distance from AoV (%)	79.22	5.51	67.03	84.57	81.69	75.42	82.96
LMCA distance from STJ (mm)	0.30	0.19	0.00	0.50	0.30	0.16	0.50
LMCA distance from STJ (mm/m^2^)	0.19	0.14	0.00	0.42	0.17	0.07	0.28
LMCA distance from STJ (%)	1.65	1.07	0.00	3.18	1.70	0.74	2.57
LMCA diameter (mm)	3.52	0.97	2.64	5.50	3.15	2.74	4.43
LMCA emergence degree (°)	131.15	12.20	113.60	152.60	130.55	121.23	140.28
LMCA above the STJ							
LMCA distance from AoV (mm)—oblique	13.24	3.61	5.20	19.60	13.60	12.44	15.39
LMCA distance from AoV (mm/m^2^)	11.68	3.02	3.10	16.14	12.02	10.59	13.08
LMCA distance from AoV (%)	90.35	17.50	28.71	104.00	96.48	86.34	98.65
LMCA distance from STJ (mm)	−3.77	7.69	−33.40	−0.10	−2.10	−2.86	−1.20
LMCA distance from STJ (mm/m^2^)	−2.65	4.30	−17.87	−0.13	−1.74	−2.34	−0.90
LMCA distance from STJ (%)	−23.81	38.58	−165.35	−0.73	−14.29	−17.87	−9.03
LMCA diameter (mm)	5.44	10.96	1.50	47.80	2.60	2.35	3.05
LMCA emergence degree (°)	129.34	18.45	94.40	164.00	131.40	114.00	141.55

AoV = aortic valve; STJ = sinotubular junction; RCA = right coronary artery; LMCA = left main coronary artery.

## Data Availability

The data presented in this study are available on request from the corresponding author.

## Supplementary Materials

The following supporting information can be downloaded at: https://www.mdpi.com/article/10.3390/healthcare10101890/s1, Table S1: Pearson correlation coefficients; Table S2: Predicted values (Mean ± 2SD) of measured echocardiography variables expressed by body surface area (BSA) (Haycock); Table S3: Predicted values (Mean ± 2SD) of measured echocardiography variables expressed by body surface area (BSA) (Haycock); Table S4: Inter- and intra-observer reliability analysis. CV, coefficient of variation.
