# Supplementary material for: Screening of Coronary Artery Origin by Echocardiography: Definition of Normal (and Abnormal) Take-Off by Standard Echocardiographic Views in a Healthy Pediatric Population
Source: Healthcare (Basel). 2022 Sep 28;10(10):1890. doi: 10.3390/healthcare10101890 (PMC9601645; doi:10.3390/healthcare10101890)
Supplement: Supplementary file 1 [file healthcare-10-01890-s001.zip › healthcare-1874154-supplementary.pdf]

Supplemental tables

Supplemental Table S1: Pearson correlation coefficients.

Measurements	*Age (years)*		*BSA*		*Weight (kg)*		*Height (cm)*	
	Pearson	***p***	***Pearson***	***p***	***Pearson***	***p***	***Pearson***	***p***
RCA distance from AoV (mm) - Oblique	0.591**	<0.0001	0.681**	<0.0001	0.650**	<0.0001	0.690**	<0.0001
RCA distance from AoV (mm) - Horizontal	0.547**	<0.0001	0.648**	<0.0001	0.617**	<0.0001	0.661**	<0.0001
LMCA distance from AoV (mm) - Oblique	0.403**	<0.0001	0.433**	<0.0001	0.419**	<0.0001	0.423**	<0.0001
LMCA distance from AoV (mm) - Horizontal	0.394**	<0.0001	0.429**	<0.0001	0.416**	<0.0001	0.420**	<0.0001
RCA Distance to STJ	0.135**	<0.0001	0.105**	0.006	0.105**	0.006	0.095*	0.013
LMCA Distance to STJ	0.338**	<0.0001	0.326**	<0.0001	0.312**	<0.0001	0.332**	<0.0001
RCA degree	-0.066	0.09	-0.110**	0.005	-0.113**	0.003	-0.091*	0.019
LMCA degree	0.164**	<0.0001	0.168**	<0.0001	0.173**	<0.0001	0.139**	<0.0001

***. Correlation is significant at the 0.01 level (2-tailed).*

**. Correlation is significant at the 0.05 level (2-tailed).*

AoV=aortic valve; BSA= Body Surface Area; LAD= Left anterior descending artery; STJ= Sino-tubular Junction.

RCA=right coronary artery; LMCA=left main coronary artery

Supplemental Table S2: Predicted values (Mean ±2SD) of measured echocardiography variables expressed by body surface area (BSA) (Haycock).

	**0.10**	**0.15**	**0.20**	**0.25**	**0.30**	**0.35**	**0.40**	**0.50**	**0.60**
	1.37	1.72	2.03	2.31	2.57	2.80	3.03	3.44	3.82
RCA distance from AoV (mm) – Oblique	**2.49**	**3.14**	**3.71**	**4.21**	**4.67**	**5.11**	**5.51**	**6.26**	**6.95**
	4.54	5.73	6.75	7.67	8.52	9.30	10.04	11.41	12.67
	1.18	1.49	1.76	2.00	2.22	2.42	2.61	2.97	3.30
RCA distance from AoV (mm) – Horizontal	**2.34**	**2.96**	**3.48**	**3.96**	**4.39**	**4.80**	**5.18**	**5.89**	**6.53**
	4.65	5.86	6.91	7.85	8.71	9.51	10.27	11.66	12.95
	0.55	0.68	0.80	0.90	0.99	1.08	1.16	1.31	1.44
LMCA distance from AoV (mm) – Oblique	**1.26**	**1.57**	**1.83**	**2.06**	**2.27**	**2.47**	**2.65**	**2.99**	**3.29**
	2.89	3.59	4.18	4.72	5.20	5.65	6.07	6.84	7.54
	0.53	0.65	0.76	0.86	0.95	1.03	1.10	1.24	1.37
LMCA distance from AoV (mm) – Horizontal	**1.20**	**1.49**	**1.73**	**1.95**	**2.15**	**2.34**	**2.51**	**2.83**	**3.11**
	2.72	3.37	3.93	4.43	4.88	5.30	5.70	6.42	7.07

AoV=aortic valve, RCA=right coronary artery; LMCA=left main coronary artery

Supplemental Table S3: Predicted values (Mean ±2SD) of measured echocardiography variables expressed by body surface area (BSA) (Haycock).

	0.7	0.8	0.9	1.0	1.1	1.2	1.3	1.4	1.5	1.6	1.7
	4.17	4.50	4.81	5.11	5.40	5.68	5.94	6.20	6.45	6.69	6.93
RCA distance from AoV (mm) – Oblique	**7.60**	**8.20**	**8.77**	**9.32**	**9.84**	**10.34**	**10.83**	**11.30**	**11.76**	**12.20**	**12.63**
	13.84	14.94	15.98	16.98	17.93	18.85	19.73	20.59	21.42	22.23	23.01
	3.60	3.89	4.16	4.41	4.66	4.90	5.13	5.35	5.57	5.78	5.98
RCA distance from AoV (mm) – Horizontal	**7.13**	**7.70**	**8.24**	**8.75**	**9.24**	**9.71**	**10.17**	**10.61**	**11.03**	**11.45**	**11.85**
	14.14	15.26	16.33	17.34	18.31	19.25	20.15	21.02	21.87	22.69	23.49
	1.56	1.68	1.79	1.89	1.99	2.09	2.18	2.27	2.35	2.43	2.52
LMCA distance from AoV (mm) – Oblique	**3.58**	**3.84**	**4.09**	**4.33**	**4.56**	**4.78**	**4.99**	**5.19**	**5.38**	**5.57**	**5.76**
	8.19	8.80	9.37	9.91	10.43	10.93	11.41	11.87	12.32	12.75	13.18
	1.49	1.60	1.70	1.80	1.90	1.99	2.07	2.16	2.24	2.32	2.39
LMCA distance from AoV (mm) – Horizontal	**3.38**	**3.63**	**3.87**	**4.09**	**4.31**	**4.51**	**4.71**	**4.90**	**5.08**	**5.26**	**5.43**
	7.68	8.25	8.78	9.29	9.78	10.24	10.69	11.12	11.54	11.94	12.33

AoV=aortic valve, RCA=right coronary artery; LMCA=left main coronary artery

Supplemental Table S4: Inter- and intra-observer reliability analysis. CV, coefficient of variation.

Measurements	***ICC Inter-observer***	***ICC Intra-observer***	***CV Inter-observer***	***CV Intra-observer***
RCA height position from AoV (mm)	0.855 (0.798-0.889)	0.870 (0.782-0.949)	2.9%	3.2%
RCA emergency angle (°)	0.788 (0.731-0.851)	0.793 (0.755-0.845)	7.1%	5.3%
LMCA height position from AoV (mm)	0.934 (0.873-0.948)	0.824 (0.791-0.937)	5.7%	7.5%
LMCA emergency angle (°)	0.772 (0.729-0.819)	0.836 (0.648-0.889)	8.4%	6.3%
AoV Diameter (mm)	0.896 (0.789-0.960)	0.892 (0.819-0.911)	4.5%	4.8%
Aortic Root Diameter (mm)	0.872 (0.758-0.939)	0.997 (0.996-0.999)	3.6%	3.3%
Aortic STJ (mm)	0.772 (0.728-0.819)	0.839 (0.748-0.889)	5.4%	3.2%
AoV-STJ distance (mm)	0.902 (0.792-0.947)	0.826 (0.781-0.937)	4.6%	7.5%

AoV=aortic valve; BSA= Body Surface Area; CFx= left circumflex artery; *LAD= Left anterior descending artery; STJ= Sino-tubular Junction; RCA=right coronary artery; LMCA=left main coronary artery, ICC=interclass coefficient, CV= coefficient of variation*
